# Spatial analysis of ischemic stroke in Spain: the roles of accessibility to healthcare and economic development

**DOI:** 10.1590/0102-311XEN212923

**Published:** 2024-09-20

**Authors:** Carlos Marcelo Leveau, Javier Riancho, Jeffrey Shaman, Ana Santurtún

**Affiliations:** 1 Consejo Nacional de Investigaciones Científicas y Técnicas, Buenos Aires, Argentina.; 2 Instituto de Producción, Economía y Trabajo, Universidad Nacional de Lanús, Remedios de Escalada, Argentina.; 3 Hospital Sierrallana, Universidad de Cantabria, Santander, España.; 4 Universidad de Cantabria, Santander, España.; 5 Instituto de Investigación Marqués de Valdecilla, Santander, España.; 6 Columbia University, New York, U.S.A.

**Keywords:** Stroke, Health Services Accessibility, Economic Factors, Mortality, Patient Admission, Accidente Cerebro Vascular, Accesibilidad a los Servicios de Salud, Factores Económicos, Mortalidad, Admisión del Paciente, Acidente Vascular Cerebral, Acessibilidade aos Serviços de Saúde, Fatores Econômicos, Mortalidade, Admissão do Paciente

## Abstract

Ischemic stroke is a major cause of mortality worldwide; however, few studies have been conducted to measure the impact of the distribution of healthcare services on ischemic stroke fatality. This study aimed to explore the relationship between three ischemic stroke outcomes (incidence, mortality, and fatality) and accessibility to hospitals in Spain, considering its economic development. A cross-sectional ecological study was performed using data on hospital admissions and mortality due to ischemic stroke during 2016-2018. Gross geographic product (GGP) per capita was estimated and a healthcare accessibility index was created. A Besag-York-Mollié autoregressive spatial model was used to estimate the magnitude of association between ischemic stroke outcomes and economic development and healthcare accessibility. GGP per capita showed a geographical gradient from southwest to northeast in Spain. Mortality and case-fatality rates due to ischemic stroke were higher in the south of the country in both women and men aged 60+ years. In women and men aged 20-59 years a EUR 1,000 increase in GGP per capita was associated with decreases in mortality of 5% and 4%, respectively. Fatality decreased 3-4% with each EUR 1,000 increase of GGP per capita in both sexes and in the 20-59 and 60+ age groups. Decreased healthcare accessibility was associated with higher fatality in the population aged 60+. Economic development in southwest Spain would not only improve employment opportunities but also reduce ischemic stroke mortality. New health related strategies to improve hospital accessibility should be considered in more sparsely populated regions or those with worse transport and/or healthcare infrastructure.

## Resumen

El ictus isquémico es una de las principales causas de mortalidad en todo el mundo; sin embargo, pocos estudios han medido el impacto de la distribución de los servicios de salud sobre la letalidad del ictus isquémico. Este estudio exploró la relación entre tres desenlaces del ictus isquémico (incidencia, mortalidad y letalidad) y la accesibilidad a los hospitales en España, teniendo en cuenta el desarrollo económico. Se realizó un estudio ecológico transversal utilizando datos que captan todas las hospitalizaciones y la mortalidad por ictus isquémico durante el período 2016-2018. Se calculó el producto geográfico bruto (PGB) per cápita y se creó un índice de accesibilidad a la salud. Se utilizó un modelo espacial autorregresivo de Besag-York- Mollié para estimar la magnitud de la asociación entre los desenlaces del ictus isquémico y el desarrollo económico y la accesibilidad a la salud. El PGB per cápita mostró un gradiente geográfico de suroeste a noreste en España. Las tasas de mortalidad y letalidad por ictus isquémico fueron mayores en el sur del país, tanto en mujeres como en hombres mayores de 60 años. En mujeres y hombres de 20 a 59 años, un aumento de EUR 1.000 en el PGB per cápita se asoció con una disminución en la mortalidad del 5% y del 4%, respectivamente. La letalidad disminuyó 3-4% por cada EUR 1.000 de aumento del PGB per cápita en ambos sexos y en los rangos de edad de 20-59 y mayores de 60 años. La disminución del acceso a la salud se asoció con una mayor mortalidad en la población mayor de 60 años. El desarrollo económico en el suroeste de España no solo mejoraría las oportunidades de empleo, sino que también reduciría la mortalidad por ictus isquémico. Se deben considerar nuevas estrategias relacionadas con la salud para mejorar la accesibilidad hospitalaria en regiones menos pobladas o con peor infraestructura de transporte o salud.

## Resumo

O acidente vascular cerebral isquêmico (AVC) é uma das principais causas de mortalidade no mundo; no entanto, poucos estudos têm mensurado o impacto da distribuição dos serviços de saúde sobre a letalidade do AVC. Este estudo explorou a relação entre três desfechos do AVC (incidência, mortalidade e letalidade) e a acessibilidade à hospitais na Espanha, considerando o desenvolvimento econômico. Um estudo ecológico transversal foi realizado usando dados que capturam todas as internações e mortalidade por AVC durante 2016-2018. Calculou-se o produto geográfico bruto (PGB) *per capita* e criou-se um índice de acessibilidade à saúde. Um modelo espacial autorregressivo de Besag-York- Mollié foi utilizado para estimar a magnitude da associação entre os desfechos do AVC e o desenvolvimento econômico e a acessibilidade à saúde. O PGB *per capita* mostrou um gradiente geográfico de sudoeste para nordeste na Espanha. As taxas de mortalidade e letalidade por AVC foram maiores no sul do país, tanto em mulheres quanto em homens com mais de 60 anos. Em mulheres e homens com idades entre 20 e 59 anos, um aumento de EUR 1.000 no PGB *per capita* foi associado a diminuições na mortalidade de 5% e 4%, respetivamente. A letalidade diminuiu 3-4% a cada aumento de EUR 1.000 no PGB *per capita* em ambos os gêneros e nas faixas etárias de 20-59 e 60+. A diminuição do acesso à saúde foi associada à maior letalidade na população 60+. O desenvolvimento econômico no sudoeste da Espanha não só melhoraria as oportunidades de emprego, mas também reduziria a mortalidade devido ao AVC. Novas estratégias relacionadas à saúde devem ser consideradas para melhorar a acessibilidade hospitalar em regiões menos povoadas ou com pior infraestrutura de transporte e/ou saúde.

## Introduction

Stroke is one of the leading causes of morbidity and mortality in industrialized countries, the second largest cause of death globally (after ischemic heart disease), and a major cause of disability worldwide ^1^ ^,^ ^2^. This illness is a multifactorial disorder, and some authors have suggested that 80 to 90% of all strokes may be preventable with lifestyle modifications and medical measures ^3^ ^,^ ^4^ that focus on known modifiable risk factors (such as hypertension, dyslipidemia, diabetes mellitus, alcohol consumption, physical activity, eating habits, obesity, etc.) ^5^ ^,^ ^6^. Notably, various studies have described associations between many of the modifiable risk factors for stroke and low income, and some have showed the relation between the risk of stroke and low socioeconomic status ^7^ ^,^ ^8^ ^,^ ^9^. Therefore, global stroke prevention policies may not equally affect all population groups.

In addition to the importance of primary prevention, medical intervention is essential for diminishing stroke fatality and associated sequelae. The *Global Burden of Disease Stud*y, published in 2019, found that age-standardized death rates from stroke have decreased by 36.2% from 1990 to 2016, globally ^10^. This trend can be explained by the improvement in the management of stroke: early recognition of symptoms, emergency interventional treatment (specially of acute ischemic stroke), and treatment in specialized stroke centers ^11^.

Ischemic stroke episodes comprise the highest number of stroke incidents ^2^ and most of them are due to embolism, either from atherosclerotic plaque in the aortic arch, in the cervical arteries, or from the heart ^12^. The magnitude of the reduction in cerebral blood flow and the time that elapses before its restoration will determine the severity of the injury, thus therapies to restore blood flow must be implemented as soon as possible. Cerebral infarction is the primary lesion derived from ischemic stroke. Due to inadequate supply of blood to the brain, a reversible loss of tissue function manifests initially; however, after some time, infarction with loss of neurons and supportive structures may occur ^13^.

Much progress has been made in recent years in the treatment of ischemic stroke: rapid reperfusion due to the efficacy of thrombolytic therapy (via the use of intravenous thrombolysis and endovascular thrombectomy) has led to the recovery of many patients ^12^ ^,^ ^14^.

Given that the main therapeutic goal for ischmeic stroke patients is the prompt restoration of blood flow to salvageable ischemic brain tissue not already infarcted ^14^, the possibility of quickly accessing a medical center is decisive in the evolution of the patient. Recently, a study carried out by Alloza et al. ^15^ analyzed the differences in accessibility to services between rural and urban areas in European countries and concluded that rural areas in Spain show less access to services than their European counterparts.

In Spain, an uneven process of industrialization began in the mid-19th century. Since then, the rural population has decreased from 39% of the population in the 1950s to less than 18% today. Currently, the highest levels of income and well-being are concentrated in Spain’s urban environments, housing most services. However, no studies have been carried out in the country to measure the impact of rural depopulation and the distribution of healthcare services on health ^16^.

The uneven process of industrialization has also led to economic divisions within Spain, with the north region presenting a more developed profile compared to the rest of the country. Economic development at the provincial level may act as a macro contextual factor associated with the risk of ischmeic strok incidence, mortality, and fatality, regardless of the risk factors at the individual level. Contextual factors, such as access to healthy food, public areas for physical activity, and high social cohesion, cannot only promote healthy behaviors that reduce the incidence of ischemic stroke, but also reduce post-ischemic stroke mortality ^17^. These local contextual factors could be more prevalent in more economically developed provinces.

The main goal of this study is to explore the relation between different epidemiological indicators of ischemic stroke (incidence, mortality, fatality) and accessibility to hospitals in Spain at the province (second-level administrative divisions) level, while considering the economic development of each region.

## Materials and methods

A cross-sectional ecological study to analyze the relation between hospital admissions, mortality and case fatality due to ischemic stroke, gross geographic product (GGP) per capita (province output), and healthcare accessibility index was performed. The geographical units of analysis were the 50 provinces in which Spain is divided. The sources of information were aggregated for the 2016-2018 period.

Municipal data is used when calculating healthcare accessibility index. Municipalities are the lowest-level territorial administrative divisions in Spain. There are a total of 8,131 municipalities in Spain. The average population of a municipality is about 5,800, but this figure masks a huge range: the most populous municipality is Madrid (capital of the province with the same name, and of Spain), with a population of 3,334,730, while several rural municipalities have fewer than 10 inhabitants. Total areas of municipalities also show large differences, with the largest municipality being Cáceres (capital of the province with the same name) with 1,750km^2^, and Emperador (province of Valencia), the smallest, with 0,03km^2^.

### Medical data

The data source used for health information was the Spanish National Institute of Statistics (INE, acronym in Spanish). Data concerning all hospital admissions and mortality due to ischemic stroke (International Classification of Diseases, 10th revision [ICD-10], codes I63 to I66 [I63: cerebral infarction; I64: stroke, not specified as hemorrhage or infarction; I65: occlusion and stenosis of precerebral arteries, not resulting in cerebral infarction; I66: occlusion and stenosis of cerebral arteries, not resulting in cerebral infarction]) were collected, including patient sex, age-group, and province. Fatality was estimated as a quotient with the number of deaths in each province as numerator and the number of hospital admissions as denominator.

### Indices

In total, two indices were used in the analysis.

GGP per capita, employed as an economic index, was provided by the INE at the provincial level. This index was employed both to evaluate its relation with incidence, mortality, and case-fatality rates, and to consider it as a confounding factor in the analysis of healthcare accessibility.

Accessibility to healthcare services was assessed in each province by defining a new index, called healthcare accessibility index (HAcI), based on the data provided by the Spanish Ministry of Health for each hospital in 2021; healthcare accessibility index is defined as:

$$HAcI=\frac{P_{nh}}{P_{t}}\times \frac{S_{nh}}{S_{t}}$$

In which:

*P*_*nh*_ is the sum of the resident population in the municipalities of the province that do not have a hospital with the minimum characteristics needed to treat ischemic stroke (defined as follows).

*P*_*t*_ is the total population of the province.

*S*_*nh*_ is the sum of the area of those municipalities of the province that do not have a hospital trained to treat ischemic stroke.

*S*_*t*_ is the total area of the province.

This index was created to account for the surface and population characteristics of provinces and municipalities in Spain. Healthcare accessibility index holds higher scores for provinces with larger percentages of population and/or area without hospitals in the municipality, so that:

• Provinces in which the population live mostly in municipalities with hospitals score lower;

• Provinces in which the population is more dispersed (either because a large proportion live outside municipalities with hospitals or because there are large areas without hospitals, or both) score higher;

• If two provinces have a similar population distribution, the one with a larger proportional area without hospitals will score higher. Similarly, if two provinces have the same ratio of area without hospitals, the one with a more scattered population will score higher.

To gauge which municipalities presented a health service capable of caring for a patient with an ischemic stroke, this study only included those that showed: (1) at least one center classified as a general hospital; (2) at least 100 beds; and (3) equipment for nuclear magnetic resonance (NMR) imaging.

Population and area data for each municipality were obtained from the Spanish National Center for Geographic Information (CNIG, acronym in Spanish).

### Statistical analysis

Ecological regressions were performed using the rate of hospital admissions, mortality, and fatality as dependent variables, stratified by sex and age groups (females and males of 20-59 and 60+ years). This stratification was adopted due to an abrupt increase in the mortality rate from ischemic stroke after the age of 60, as well as a higher mortality rate in males ^18^. GGP per capita and HAcI were used as explanatory variables. For each outcome, a Poisson Besag-York-Mollié (BYM) model was fitted ^19^. These multivariate models showed a better fit, compared to the models assuming negative binomial distribution (Supplementary Material - Table S1: https://cadernos.ensp.fiocruz.br/static//arquivo/suppl-e212923_1051.pdf). BYM models are part of Bayesian methods that incorporate a hierarchical structure, allowing considering similarities based on neighborhood relationships ^20^. Taking advantage of aggregated data in georeferenced areas, this model includes both nonspatial and spatial random effects. Spatially structured random effects were estimated considering a spatial contiguity matrix, in which the neighboring province was assumed to be defined for the first neighbor only, defined by common boundary. The BYM model was reparametrized as proposed by Riebler et al. ^21^, known as the BYM2 model, and assigned penalized complexity priors as hyperparameters ^22^. Models with hospital admissions and mortality as outcomes included population of specific sex and age groups as offset, whereas models with fatality as outcomes included hospital admissions of specific sex and age groups as offset. As a first step, unadjusted models were developed including one explanatory variable. Then, adjusted models were fitted including both explanatory variables. Finally, the precision for the random effect was computed (sum of structured plus unstructured effects), as well as the Phi for ID (the index variable of each area), which measures the proportion of the marginal variance explained by the structural effect.

The spatial regression models were conducted using the R, version R 3.2.5 (http://www.r-project.org), using the INLA package. The mapping of the variables was carried out with the QGIS program, version 2.14.3 (https://qgis.org/en/site/).

## Results

**Figure 1:**
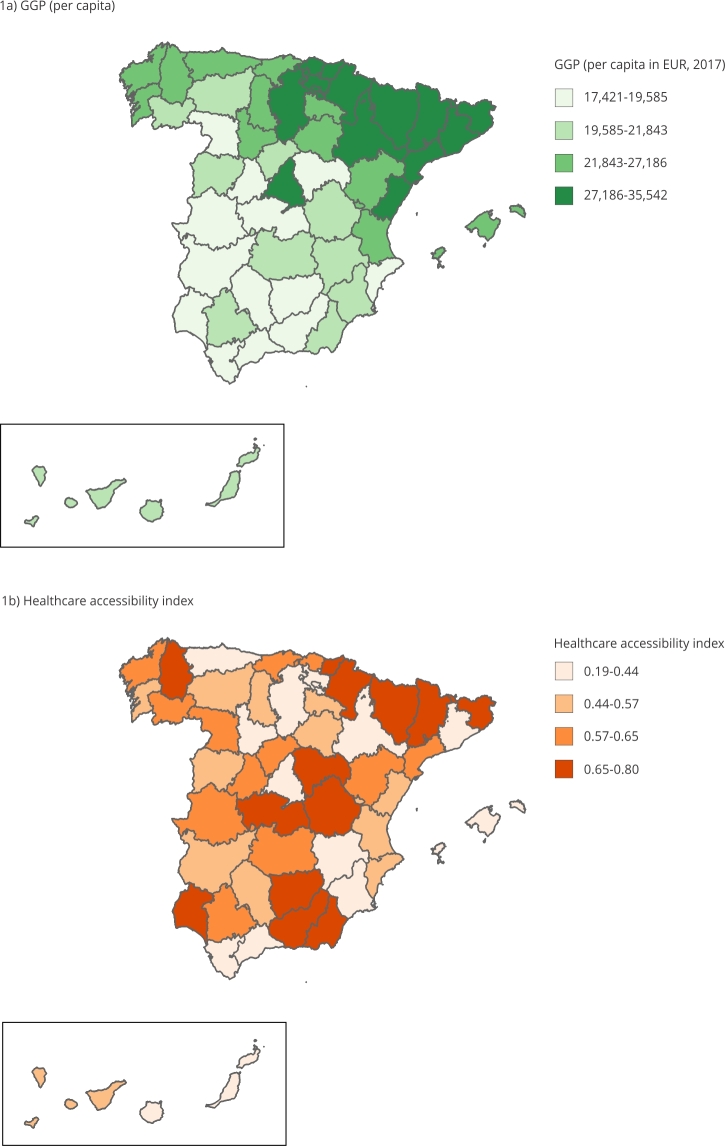
Geographical distribution of gross geographic product (GGP) per capita and the healthcare accessibility index in Spain.

Figure 1 shows the geographical distribution of the GGP per capita and the accessibility index. While the GGP per capita shows a pattern of north-south economic development, the accessibility index mainly shows three areas of low accessibility: in the Southeastern Spain; around the province of Madrid; and in the Northeastern Spain.

From 2016 to 2018, 209,799 hospital admissions for ischemic stroke were recorded in Spain, and the overall mean incidence rate was 150.17 admissions/100,000 population. In the three years analyzed, 48,426 people died in the country from ischemic stroke. Females comprised 45.25% of registered admissions but 60.92% of deaths. Average age of death from ischemic stroke was 82 years in males and 87 in females.

**Figure 2:**
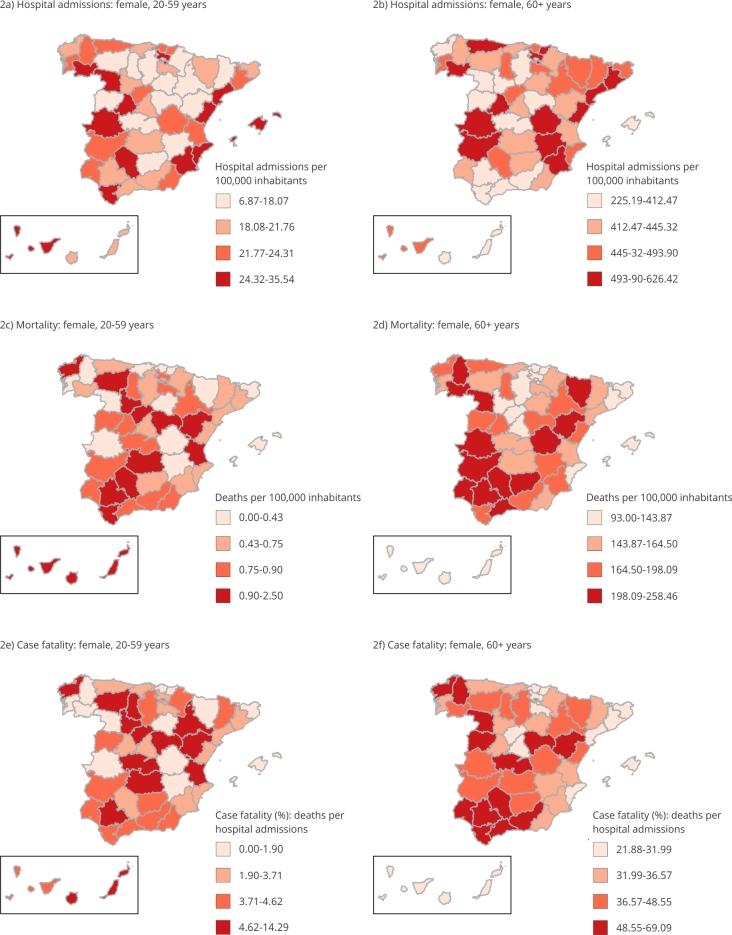
Geographical distribution of ischemic stroke outcomes in the female population of Spain, 2016-2018.

**Figure 3:**
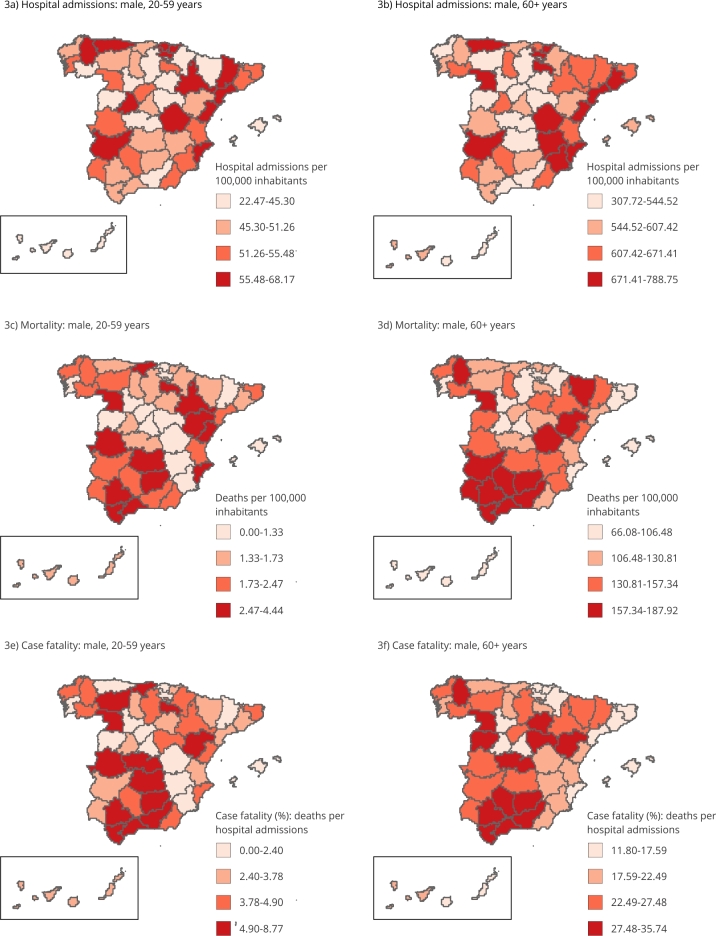
Geographical distribution of ischemic stroke outcomes in the male population of Spain, 2016-2018.

The spatial patterns of hospital admissions, mortality, and fatality due to ischemic stroke in females and males aged 60+ years are very similar (Figures 2 and 3). Mortality and case-fatality rates due to ischemic stroke show a geographic concentration of high rates in Southern Spain in both sexes.

Unlike the population aged 60+, there are differences in the geographical distributions between sexes in the population aged 20-59 years. While mortality and case-fatality rates in females are higher in the inland provinces around Madrid (Figure 2), the rates in males show a heterogeneous spatial pattern (Figure 3).

Table 1 shows relative risks (and 95% credibility intervals) of the ischemic stroke outcomes associated with GGP per capita and the healthcare accessibility index. In the unadjusted models, a EUR 1,000 increase in GGP per capita was associated with a 3-4% decrease in mortality due to ischemic stroke in both sexes and age groups (Table 1). These inverse associations prevailed in the adjusted models for patients aged 20-59 years (decreases in mortality of 5% and 4% per 1,000 per capita of increase in GGP per capita, respectively). In the adjusted models, fatality decreased 3-4% with each increase of EUR 1,000 in GGP per capita, in both sexes and age groups (Table 1).

**Table 1:** Relative risks (RR) of three ischemic stroke outcomes associated with gross geographic product (GGP) per capita and the healthcare accessibility index in Spain, 2016-2018.

	GGP per capita * RR (95%CI)		Accessibility index RR (95%CI)		Random effect (95%CI) **	Phi for ID (95%CI) **
	Unadjusted models	Adjusted models ***	Unadjusted models	Adjusted models ***		
Hospital admissions						
Female (years)						
20-59	1.01 (0.99-1.02)	1.00 (0.99-1.02)	0.67 (0.43-1.04)	0.68 (0.42-1.09)	26.19 (13.81-45.53)	0.13 (0.01-0.51)
60+	1.01 (1.00-1.02)	1.01 (1.00-1.02)	0.81 (0.57-1.16)	0.91 (0.62-1.32)	34.11 (21.52-50.72)	0.10 (0.00-0.38)
Male (years)						
20-59	1.01 (1.00-1.02)	1.01 (0.99-1.02)	0.81 (0.57-1.15)	0.87 (0.60-1.26)	37.60 (21.92-59.80)	0.14 (0.01-0.52)
60+	1.01 (1.00-1.02)	1.01 (1.00-1.02)	0.78 (0.55-1.09)	0.86 (0.61-1.23)	37.70 (23.64-56.30)	0.14 (0.01-0.47)
Deaths						
Female (years)						
20-59	0.96 (0.94-0.98)	0.95 (0.93-0.98)	1.09 (0.43-2.59)	0.51 (0.20-1.30)	950.91 (12.35-6373.93)	0.34 (0.02-0.89)
60+	0.97 (0.96-0.99)	0.98 (0.96-1.00)	1.74 (1.17-2.61)	1.42 (0.92-2.22)	22.50 (13.07-35.74)	0.50 (0.04-0.96)
Male (years)						
20-59	0.96 (0.94-0.98)	0.96 (0.94-0.99)	1.72 (0.76-3.81)	1.18 (0.55-2.52)	26.34 (7.29-76.71)	0.32 (0.01-0.89)
60+	0.97 (0.96-0.99)	0.98 (0.96-1.00)	1.71 (1.13-2.62)	1.39 (0.89-2.17)	24.68 (14.77-38.37)	0.33 (0.02-0.89)
Fatality ^#^						
Female (years)						
20-59	0.96 (0.94-0.98)	0.96 (0.93-0.98)	1.41 (0.53-3.50)	0.70 (0.26-1.87)	497.63 (8.36-3301.26)	0.32 (0.02-0.88)
60+	0.96 (0.95-0.98)	0.97 (0.95-0.99)	2.22 (1.47-3.38)	1.69 (1.07-2.65)	21.39 (13.01-32.81)	0.42 (0.05-0.88)
Male (years)						
20-59	0.95 (0.93-0.97)	0.96 (0.93-0.98)	2.09 (0.95-4.53)	1.33 (0.67-2.66)	52.19 (9.87-194.03)	0.31 (0.01-0.88)
60+	0.96 (0.94-0.98)	0.97 (0.95-0.98)	2.21 (1.44-3.41)	1.63 (1.06-2.51)	25.95 (15.58-40.35)	0.31 (0.02-0.82)

95%CI: 95% confidence interval.

* EUR 1,000 (2017);

** Computed for adjusted models;

*** Adjusted for both indicators simultaneously;

^#^ Deaths as a dependent variable and hospital admissions included as an offset in the model.

For the accessibility index, in the unadjusted models, positive associations were found with mortality and fatality in the population aged 60+ (Table 1). In the adjusted models, decreased accessibility was associated with higher fatality in the population aged 60+. After considering the GGP per capita and the healthcare accessibility index, the proportion of variance attributed to the spatial effect (Phi for ID) showed its highest values in the ischemic stroke mortality models (Table 1).

## Discussion

Ischemic stroke, similar to other cardiovascular diseases, shows a wide range of potentially modifiable risk factors, including high blood pressure, dyslipidemia, smoking and atrial fibrillation, among others ^23^. Without neglecting the importance of these “typical” conditions in the genesis of ischemic stroke, over the last decade, the role of other sociodemographic factors has been progressively assessed. On this basis, it has been recently reported that lower income and educational level were significantly associated with increased risk of ischemic stroke in the United States’ population ^24^. In addition, it has been suggested that racial inequalities also influence rehabilitation therapy in patients suffering from ischemic stroke, thus conditioning their functional recovery ^25^. However, other important sociodemographic factors, such as population dispersion or hospital accessibility, have yet to be assessed. This study was conceived to evaluate the effect of the accessibility to hospitals in Spain and the GGP per capita at the province (second-level administrative division) level on incidence, mortality, and fatality due to ischemic stroke.

Ischemic stroke incidence rate, assessed by hospital admissions, varies across Spanish provinces, exhibiting mild predominance in the south, though no clear consistent pattern. This gradient might be related to several distinct conditions, such as a higher prevalence of cardiovascular risk factors like higher blood pressure and obesity rates in southern Spanish provinces ^26^ ^,^ ^27^.

In our analysis of ischemic stroke incidence and GGP per capita, we found a significant association in the unadjusted models that becomes not significant in the final model (when accounting for accessibility to healthcare services). Several studies have suggested an inverse association between income and ischmeic stroke ^24^ ^,^ ^28^. Feigin et al. ^29^ performed a systematic review to study worldwide stroke incidence and concluded that overall stroke incidence rates in low-to-middle-income countries exceeded the level of stroke incidence seen in high-income countries by 20% from 2000 to 2008. However, our results could be explained by the hypothesis suggested by Avan et al. ^30^, who analyzed the trend in global age-standardized stroke and socioeconomic status, and found that stroke prevalence had increased in upper-middle-income countries and decreased in low-income countries. The authors suggested that the increase in stroke prevalence could partly be a result of improved healthcare and general awareness, which had extended the lifespan of stroke patients. Bearing in mind that people who have had a first stroke show a higher risk of suffering a second one ^31^, this could also explain why, when we include the healthcare accessibility index in our analysis, the relationship between ischemic stroke and GGP per capita ceases to be significant.

This study of the relationship between GGP per capita and mortality and case-fatality rates evidenced a significant inverse correlation in young adults and in all groups, respectively. In the literature, previous studies have shown that poorer territories held both higher mortality and case-fatality rates ^24^. This might be explained by several facts, including less developed health strategies, higher hospital occupation, or higher ratios of healthcare professionals (physicians, nurses) per patient ^32^.

At this point, we consider of particular interest the subsequent analysis in which we correlated mortality and fatality with accessibility. Fatality, which reflects the proportion of admitted patients with a diagnosis of ischemic stroke who died during their stay at hospital, was especially higher (more than double) in provinces with less infrastructure. Patients who live in a less developed province with reduced hospital accessibility may experience delays in receiving care. These patients may not be candidates for revascularization strategies, thus developing massive infarcts and consequently poorer survival rates. Specifically, massive strokes are associated with both neurological (malignant infarcts) and non-neurological complications (respiratory infections, cutaneous ulcers, etc.) ^33^.

Stroke revascularization strategies include both intravenous thrombolysis and mechanical thrombectomy. While intravenous thrombolysis can be administrated within the first 4.5 hours since the onset of symptoms and is available in most hospitals, mechanical thrombectomy can only be performed in some situations during the first 24 hours but is commonly centralized in reference centers of provinces ^23^. Thus, in those provinces with a more dispersed population and/or less infrastructure, patients with ischemic stroke would not only take longer to arrive to the hospital but any inter-hospital transfer would also be delayed.

Our study holds numerous strengths: this is the first study conducted in the country (and, to our knowledge, in Europe) on accessibility to health services and ischemic stroke. It proposes a new index that can be easily applied in other places if information from hospitals throughout the country is available. Furthermore, the area of analysis may be representative (Spain is the second largest country in the European Union). However, some limitations must be noted. First, the spatial units used in this study may reflect a broad level of generalization that masks significant variations within the spatial units. This is part of the modifiable area unit problem. Second, we could not introduce explanatory variables related to lifestyle, such as hypertension, dyslipidemia, obesity, diabetes mellitus, physical activity, and alcohol consumption. Third, hospital admission data did not include individual identifiers, so it was not possible to determine if the same individual had more than one ischemic stroke during 2016-2018.

## Conclusions

Our study shows a clear inverse correlation between fatality due to ischemic stroke and accessibility to health services across the Spanish provinces, potentially highlighting the effect of delays in receiving care. Based on this, new strategies to improve hospital accessibility should be considered in those regions more sparsely populated or with worse transport and/or healthcare infrastructure.
