# Supplementary material for: Spatial analysis of ischemic stroke in Spain: the roles of accessibility to healthcare and economic development
Source: Cad Saude Publica. 2024 Sep 20;40(9):e00212923. doi: 10.1590/0102-311XEN212923 (PMC11415051; doi:10.1590/0102-311XEN212923)
Supplement: Supplementary file 1 [file 1678-4464-csp-40-09-EN212923-s.pdf]

Supplementary material

Table S1 Values of the Watanabe-Akaike information criterion (WAIC) comparing "BYM2" models under Poisson distributions and negative binomial distributions.

Outcomes/Sex, age group	Poisson	Negative binomial
**Hospital admissions**		
Female (years)		
20-59	418.08	466.93
60+	528.37	671.82
Men (years)		
20-59	456.19	528.60
60+	535.06	676.88
**Deaths**		
Female (years)		
20-59	190.26	191.71
60+	479.19	562.65
Men (years)		
20-59	282.44	285.33
60+	457.74	540.73
**Fatality**		
Female (years)		
20-59	205.32	206.54
60+	479.24	557.86
Men (years)		
20-59	280.17	280.57
60+	458.61	527.59

Note: multivariate models including gross geographic product (GGP) per capita and the healthcare accessibility index in Spain, 2016-2018.
